# Association between 3D membranous urethral parameters and urinary continence recovery after RARP

**DOI:** 10.1186/s40001-024-01758-y

**Published:** 2024-03-12

**Authors:** Ming Wang, Ruiqi Deng, Lei Wang, Mingzhao Li, Tao Zeng, Yanqun Na, Di Gu

**Affiliations:** 1 Department of Urology, The First Affiliated Hospital of Guangzhou Medical University, Guangzhou, China; 2 Wujieping Urology Center, Peking University Shougang Hospital, Beijing, China

**Keywords:** Robotic-assisted radical prostatectomy, Urinary continence, Membranous urethral length, Membranous urethral complex volume, Three-dimensional model

## Abstract

**Objectives:**

To evaluate whether the urinary continence (UC) recovery after robotic-assisted radical prostatectomy (RARP) relates to the membranous urethral length (MUL) and the membranous urethral complex volume (MUV).

**Materials and methods:**

120 patients who underwent RARP were enrolled according to the different times of UC recovery and examined using prostate magnetic resonance imaging (MRI) before surgery. The membranous urethral (MU) parameters were measured using the three-Dimensional (3D) model reconstructed by holographic technology, such as total MUV (tMUV), exposed MUV (eMUV), full MUL (fMUL) and exposed MUL (eMUL). Statistical software SPSS 26.0 was used to analyze the data and compare the MU parameters and baseline data in different groups.

**Results:**

Patients with larger tMUV (*p* = 0.038), eMUV (*p* = 0.003), longer fMUL (*p* = 0.025), eMUL (*p* = 0.044) had better UC after removal of the catheter, and eMUV (OR = 1.002, 95%CI = 1.001–1.004, *p* = 0.004) was a predictor; the patients with younger age (*p* = 0.021), lower VPSS score (*p* = 0.004) and larger eMUV (*p* = 0.012) and longer eMUL (*p* = 0.049) had better UC recovery one month after RARP while eMUV (OR = 1.002, 95% CI = 1.000–1.003, *p* = 0.008) and VPSS score (OR = 0.886, 95% CI = 0.806–0.973, *p* = 0.011) were independent risk factors; The patients with younger age (*p* = 0.018), larger tMUV (*p* = 0.029), eMUV (*p* = 0.016) had better UC recovery three months after RARP. eMUV (OR = 1.002, 95% CI = 1.000–1.004, *p* = 0.042) and age (OR = 0.904, 95% CI = 0.818–0.998, *p* = 0.046) were independent risk factors.

**Conclusion:**

This clinical study shows that patients with larger MUV and longer MUL can return to UC earlier after surgery. Among that, eMUV is a better predictor.

## Introduction

Urinary incontinence is one of the major complications after radical prostatectomy (RP) [1], dramatically affecting patients’ quality of life. Research shows that over 80% of men can recover urinary continence (UC) within one year after surgery [2]. In contrast, more need two years to recover from urinary incontinence [3]. UC is the patient using ≤ 1 safety pad daily after robotic-assisted radical prostatectomy (RARP). The date on which the patient stopped using more than one safety pad was considered the recovery time of continence [4]. The recovery of UC after RARP is also affected by many factors, such as age, body mass index (BMI), comorbidity index, lower urinary tract symptoms (LUTS), and prostate volume (PV) [5]. It has been found that residual membranous urethral length (MUL) is considered an independent predictor of postoperative UC recovery, and the MUL plays a crucial role in postoperative UC [6]. There are few studies on the association between the membranous urethra (MU) and UC after RARP, all based on the magnetic resonance imaging (MRI) of prostate measurements, so the data are less accurate.

Multiparameter prostate magnetic resonance imaging (mpMRI), as the most common imaging examination of the prostate, plays a vital role in the diagnosis of prostate cancer and the decision on a surgical plan. It can help surgeons understand the anatomical structure of potential prostate cancer areas [7]. Due to the limitations of MRI, such as the two-dimensional plane and opaque, it is difficult to read without systematic training. MRI is rarely used in surgery for those reasons [8]. Recent research shows that 3-dimensional (3D) imaging has been proven to improve surgeons' understanding of patient anatomy, influence surgical planning, and improve patient outcomes in applying partial nephrectomy [9–11] and RP [12]. However, no one has studied the association between the measurement of MU parameters based on the 3D imaging reconstruction model and the UC after RARP.

Under these circumstances, we conducted a clinical study using the 3D imaging reconstruction model of preoperative prostate MRI to measure patients' MU parameters. We intend to identify the association between these parameters and the postoperative UC of patients and explore more accurate predictors of postoperative UC.

## Materials and methods

### Participant eligibility

All participants were recruited from a large teaching hospital. Patients who had undergone prostate surgery (transurethral resection of the prostate or enucleation of the prostate), did not undergo MRI examination and could not reconstruct their 3D models, or could not give informed consent were excluded from the study. Participants knew the benefits of surgery under the guidance of an individualized 3D reconstruction model and agreed that their 3D reconstruction model would be used for scientific and clinical research.

### The definition of urinary continence

All patients were followed up by telephone or outpatient after the catheter removal, one month and three months after the surgery, respectively, to evaluate their urinary situation. UC is defined as no pad or one safety pad per day, is known as social continence, which is defined as not affecting the patient's daily life. These patients were divided into different groups: social continence group, which included patients who recover to UC immediately after removal of the catheter, patients who recover to UC 1 month after surgery and patients who recover to UC 3 months after surgery, and urinary incontinence group, which refers to those who failed to recover continence in the same timeline.

### Imaging examination and 3D reconstruction model

All patients were examined with a prostate MRI before the operation. The pelvic enhanced scanning was performed with GE 3.0T Signa HDx, with a slice thickness of 2 mm. T1, T2, diffusion-weighted imaging (DWI), dynamic contrast-enhanced (DCE) sequences, and apparent diffusion coefficient (ADC) maps were obtained. MRI data were collected and saved in DICOM format. Upload MRI and diagnostic data to the holographic analysis planning system workstation (Beijing Renxin Medical Technology Co., Ltd.). After communicating with surgeons about particular concerns, especially the location of prostate cancer lesions, professional engineers will reconstruct them into holographic images based on MRI to assist in surgical planning and intraoperative navigation.

**Fig. 1:**
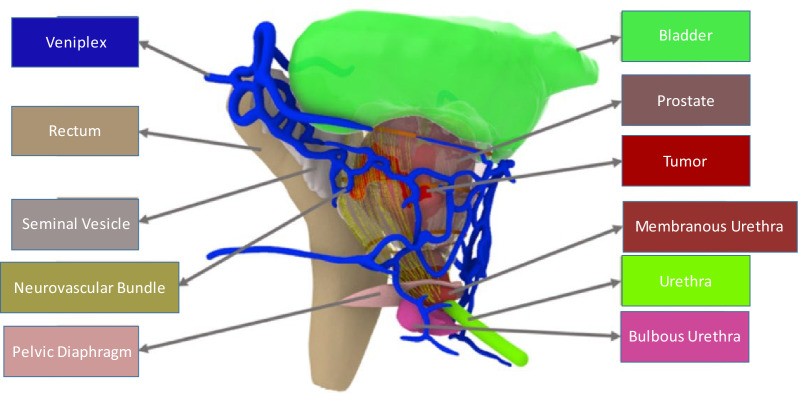
3D model reconstructed by holographic technology

**Fig. 2:**
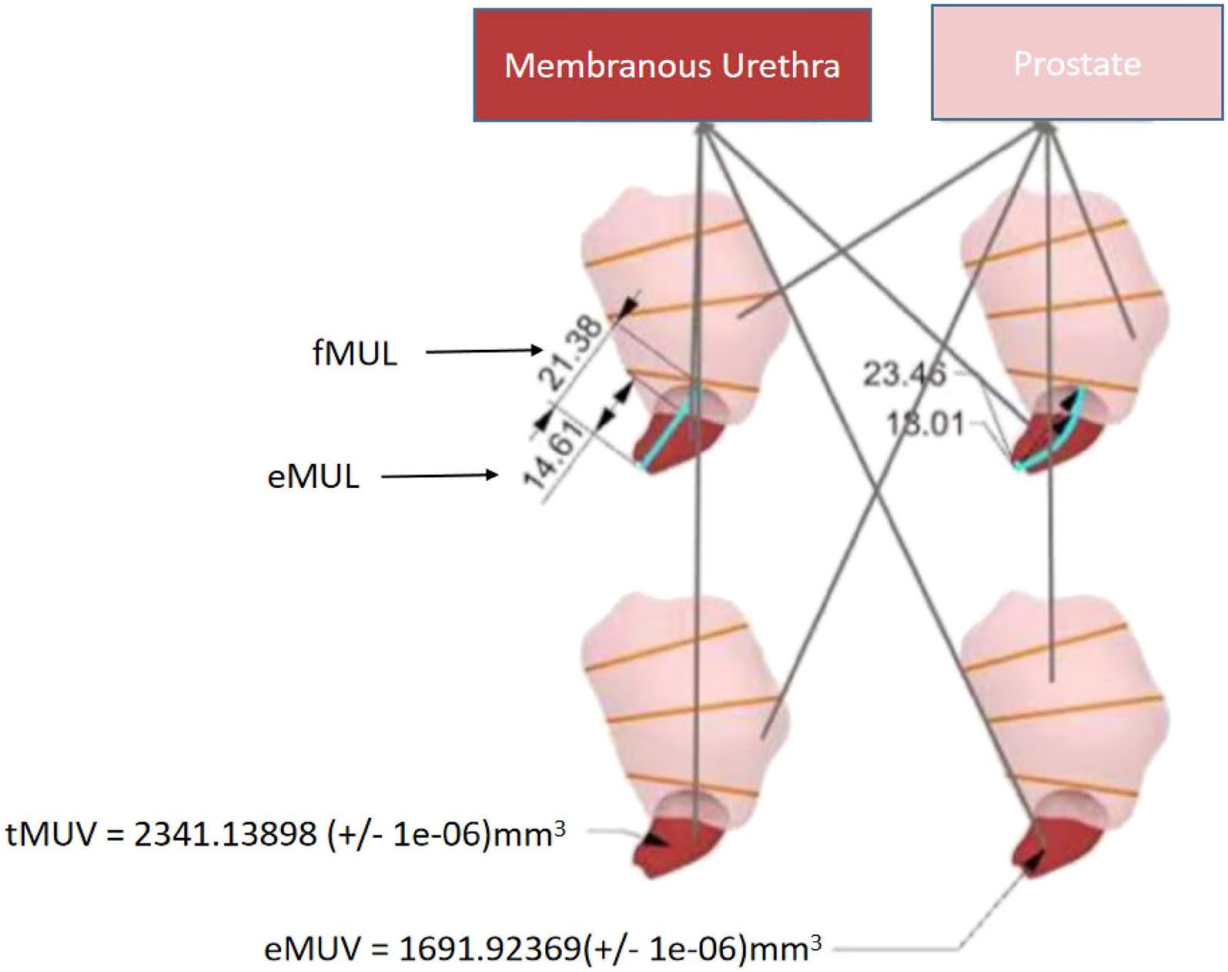
Membranous urethra reconstructed by holographic technology

The preoperative prostate MRI of patients was reconstructed into a new 3D dynamic model with multi-angle rotation, and organs can be disassembled using holographic imaging technology. The model includes the prostate, tumor, bladder, urethra, neurovascular bundle (NVB), seminal vesicle, iliac blood vessels, etc. (Fig. 1), which helps surgeons better understand the anatomical structure of patients and the location of the tumor in the prostate. The MU complex was reconstructed after the gray-scale signal mask, stacking, reconstruction, correction, and smoothing of MU in MRI data. The total volume and exposed volume of the MU complex were calculated by the Monte Carlo method, also known as the random simulation method, and the full length and exposed length of MU were measured by the Unity engine (Fig. 2). An experienced professional technician measures these parameters with the same standard.

### Surgical procedure

All operations were performed by an experienced robotic surgeon (DG). The surgeon used unified and standardized surgical techniques to operate on all the enrolled patients and used the retention techniques of UC tissue and the reconstruction techniques of UC tissue to promote the early recovery of UC.

### Statistical analysis

We use SPSS 26.0 for data analysis. To analyze the association between the baseline data of different groups, such as age, BMI, VPSS score, and our research parameters total membranous urethral complex volume (tMUV), exposed membranous urethral complex volume (eMUV), full membranous urethral length (fMUL), exposed membranous urethral length (eMUL), and PV, we first conducted a normal test on the data. We used an independent sample t-test to analyze the data that met the normality and the Mann–Whitney U test to analyze the data that did not. We took *p* < 0.05 as a statistically significant standard. We performed binary logistic regression analyses to identify independent predictive factors of UC recovery at each time point in the early postoperative stage. All variables found to be significant in the univariate analysis (*p* < 0.05) were included in the multivariate logistic regression model.

## Results

This study included 120 prostate cancer patients who received RARP in the First Affiliated Hospital of Guangzhou Medical University from December 2020 to October 2022. The control parameters were patients’ age, BMI, VPSS score, tMUV, eMUV, fMUL, eMUL, and PV. One hundred twenty patients were divided into different groups according to the time that they returned to UC. The patients achieved UC in different timelines: the achieving UC immediately after removing the catheter group, the achieving UC 1 month after surgery group, and the achieving UC 3 months after surgery group. These groups were compared with those who failed to achieve continence in different timelines. This information was collected by telephone followed up or outpatient service.

For achieving UC immediately after removing the catheter group and the control group, 27 patients were included in the achieving continence immediately after removing the catheter group, and 93 patients were included in the failed to achieve continence after removing the catheter group. The data showed that the UC rate immediately after the catheter removal was 22.5%. The baseline data, such as age, BMI, VPSS score, and PV, showed no significant difference. The mean tMUV was 1533.02 and 1299.02 mm^3^, respectively. Those with bigger tMUV had better UC recovery (*p* = 0.038); the mean eMUV was 1014.39 and 830.14 mm^3^, respectively. Those with larger eMUV had better UC recovery (*p* = 0.003). Moreover, the mean fMUL was 19.65, and 18.41 mm, respectively; the patients with longer fMUL had better UC recovery (*p* = 0.025), and the mean eMUL was 13.62 and 12.69 mm, respectively. The patients with longer eMUL had better UC recovery (*p* = 0.044). Multivariate analysis showed that eMUV (OR = 1.002, 95%CI = 1.001–1.004, *p* = 0.004) was independently associated with continence return immediately after removing the catheter (Table 1).

**Table 1:** Univariate and multivariate regression analysis of patients achieving continence immediately after removing catheter and those who cannot

	Total	Patients achieving continence immediately after removing catheter group		Patients failed to achieve continence after removing catheter group		P	Multivariate		
							OR	95%CI	P
No. of patients (%)	120	27 (22.5)		93 (77.5)					
		Mean	SD	Mean	SD				
Age (years)		67.59	6.90	69.78	5.24	0.078			
BMI (kg/m^2^)		23.21	2.61	23.47	2.62	0.546			
tMUV (mm^3^)		1533.02	466.30	1299.02	460.79	**0.038**	1.000	0.998–1.002	0.704
eMUV (mm^3^)		1014.39	267.99	830.14	280.20	**0.003**	1.002	1.001–1.004	**0.004**
fMUL (mm)		19.65	2.10	18.41	2.71	**0.025**	1.045	0.823–1.326	0.718
eMUL (mm)		13.62	2.41	12.69	2.01	**0.044**	1.003	0.998–1.008	0.247
PV (cc)		38.97	27.90	35.16	22.37	0.927			
VPSS		9.00	0.91	10.14	0.42	0.095			

The statistically significant p values in the table are shown in bold

*BMI* body mass index, *OR* odd ratio, *CI* confidence interval, *SD* standard deviation, *tMUV* total membranous urethral complex volume, *eMUV* exposed membranous urethral complex volume, *fMUL* full membranous urethral length, *eMUL* exposed membranous urethral length, *PV* prostate volume, *VPSS* visual prostate symptom score

For the achieving UC 1 month after RARP group and the failed to achieve continence 1 month after RARP group, 60 patients were included in the continence group, and 60 patients were included in the incontinence group. The data showed that the UC rate 1 month after RARP was 50%. The analysis showed that younger patients had a higher probability of resuming UC 1 month after RARP, and the results were statistically significant (*p* = 0.021). The patients with lower VPSS score had better UC recovery (*p* = 0.004). The mean eMUV was 936.91 and 806.28 mm^3^, respectively. Those with larger eMUV had better UC recovery (*p* = 0.012). The mean eMUL was 13.28 and 12.52 mm, respectively. The patients with longer eMUL had better UC recovery (*p* = 0.049); the remaining data were not statistically significant. Multivariate analysis showed that eMUV (OR = 1.002, 95% CI = 1.000–1.003, *p* = 0.008) and VPSS score (OR = 0.886, 95% CI = 0.806–0.973, *p* = 0.011) were independent risk factors affecting continence return 1 month after RARP (Table 2).

**Table 2:** Univariate and multivariate regression analysis of patients achieving continence 1 month after RARP and those who cannot

	Total	Patients achieving continence 1 month after RARP group		Patients failed to achieve continence 1 month after RARP group		P	Multivariate		
							OR	95%CI	P
No. of patients (%)	120	60 (50)		60 (50)					
		Mean	SD	Mean	SD				
Age (years)		68.10	6.09	70.48	5.04	**0.021**	0.964	0.893–1.041	0.356
BMI (kg/m^2^)		23.29	2.61	23.50	2.62	0.660			
tMUV (mm^3^)		1416.99	470.98	1286.36	464.57	0.124			
eMUV (mm^3^)		936.91	297.40	806.28	262.65	**0.012**	1.002	1.001–1.003	**0.008**
fMUL (mm)		19.01	2.58	18.37	2.65	0.065			
eMUL (mm)		13.28	2.17	12.52	2.04	**0.049**	1.090	0.872–1.363	0.450
PV (cc)		35.83	27.51	36.20	19.29	0.220			
VPSS		8.97	0.58	10.80	0.49	**0.004**	0.886	0.806–0.973	**0.011**

The statistically significant p values in the table are shown in bold

*BMI* body mass index, *OR* odd ratio, *CI* confidence interval, *SD* standard deviation, *tMUV* total membranous urethral complex volume, *eMUV* exposed membranous urethral complex volume, *fMUL* full membranous urethral length, *eMUL* exposed membranous urethral length, *PV* prostate volume, *VPSS* visual prostate symptom score

For achieving UC 3 months after RARP group and the failed to achieve continence 3 months after RARP group, 101 patients were included in the continence group, and 19 patients were included in the incontinence group. The data showed that the UC rate recovered 3 months after RARP was 84.17%. The analysis showed that the younger patients had a higher probability of resuming UC 3 months after RARP, similar to the result in achieving UC 1 month after surgery (*p* = 0.018). The mean tMUV was 1389.58 and 1150.17 mm3, respectively. Those with bigger tMUV had better UC recovery (*p* = 0.029); the mean eMUV was 898.76 and 727.20 mm3, respectively. Those with larger eMUV had better UC recovery (*p* = 0.016). Other research parameters had no statistical significance. Multivariate analysis showed that eMUV (OR = 1.002, 95%CI = 1.000–1.004, *p* = 0.042) and age (OR = 0.904, 95%CI = 0.818–0.998, *p* = 0.046) were independent risk factors affecting continence return 3 months after RARP (Table 3).

**Table 3:** Univariate and multivariate regression analysis of patients achieving continence 3 months after RARP and those who cannot

	Total	Patients achieving continence 3 month after RARP group		Patients failed to achieve continence 3 month after RARP group		P	Multivariate		
							OR	95%CI	P
No. patients (%)	120	101 (84.17)		19 (15.83)					
		Mean	SD	Mean	SD				
Age (years)		68.76	5.60	72.11	5.54	**0.018**	0.904	0.818–0.998	**0.046**
BMI (kg/m^2^)		23.29	2.52	23.92	3.04	0.337			
tMUV (mm^3^)		1389.58	438.90	1150.17	584.66	**0.029**	1.000	0.998–1.002	0.981
eMUV (mm^3^)		898.76	272.72	727.20	324.23	**0.016**	1.002	1.000–1.004	**0.042**
fMUL (mm)		18.89	2.59	17.61	2.63	0.051			
eMUL (mm)		13.06	2.20	12.06	1.47	0.060			
PV (cc)		36.52	24.23	33.37	20.70	0.570			
VPSS		9.57	0.42	11.53	0.96	0.058			

The statistically significant p values in the table are shown in bold

*BMI* body mass index, *OR* odd ratio, *CI* confidence interval, *SD* standard deviation, *tMUV* total membranous urethral complex volume, *eMUV* exposed membranous urethral complex volume, *fMUL* full membranous urethral length, *eMUL* exposed membranous urethral length, *PV* prostate volume, *VPSS* visual prostate symptom score

## Discussion

RP is currently the most suggested treatment for localized and low-risk prostate cancer [13]. The goals of RP are proposed as the term of PENTAFECTA, which mainly include effective cancer control, rapid return of UC, recovery of sexual function, positive surgical margin rates, and perioperative complications [14]. Among these goals, the most crucial goal recognized by surgeons and patients is cancer control [15], while the most needed one is UC [16]. RARP has advantages in achieving these goals and has become a new gold standard for the radical treatment of clinically localized prostate cancer [17].

With the rapid development of technology, more and more prostate cancer patients choose RARP as their preferred treatment approach. Postoperative urinary incontinence and sexual dysfunction, the most important complications after RP, significantly affect patients’ quality of life. Studies have found that the recovery of UC after RARP is affected by many factors, such as age, BMI, comorbidity index, LUTS, and PV [5], which can affect the occurrence of urinary incontinence after RARP. The incidence of urinary incontinence is also affected by surgeons’ experience, choice of surgical plans, and even statistical analysis of data [18].

In this study, we collected patients’ VPSS score before surgery to explore the relationship between LUTS and the recovery of UC after RARP. There is a strong correlation between VPSS score and IPSS score. VPSS score can be used and understood easily and is more time-saving, which applies to all education groups and is an auxiliary or alternative tool in addition to IPSS score [19]. Haga et al. found no significant correlation between preoperative and postoperative MRI variables such as MUL and posterior-urethral vesical angle [20]. Whether VPSS score or IPSS score can predict the recovery of postoperative UC in patients still needs further study.

To promote the early UC recovery of patients after RARP, researchers from all over the world keep on exploring approaches that can help with the recovery of UC. Among them, optimizing and standardizing the surgical techniques of surgeons is the most effective way to improve early UC recovery after RARP. For example, the research results of NYARANGI-DIX et al. show that the weight of urine leakage at 3, 4, and 12 months after surgery in patients receiving bladder neck preservation during RARP was lower than that in patients without bladder neck preservation [21]; REEVES et al. found through a systematic review and meta-analysis that the rate of early UC in patients with preservation of the NVB was improved 6 months after surgery compared with patients without NVB preservation [22]; SCHLOMM et al. published their surgical technique of Full functional-length urethral (FFLU) sphincter preservation in RP surgery. The results showed that patients using the FFLU technique had a higher UC rate one week after the catheter removal than those without the FFLU technique. They also emphasized preserving every millimeter of urethra [23]. Nowadays, the procedure and techniques of RARP have been standardized. We aim to help patients achieve earlier UC after surgery and improve their quality of life. However, some patients still suffer from urinary incontinence after surgery. Therefore, it is essential to find the predictors of UC after RARP.

Longer MUL has been proven to be helpful for early recovery of UC. Paparel et al. measured MUL of 64 RP patients before and after surgery with MRI and found that a longer preoperative or postoperative MUL was associated with superior continence [24]. MUNGOAN et al. reported in a systematic review and meta-analysis that every extra millimeter of MUL was associated with significantly greater odds for return to continence [25]. The research results of KO et al. also showed that, regardless of the benefit in maximizing MUL, an innate “predetermined” length of native MU less than approximately 1.4 cm reduces both 30-day and 1-year continence recovery [26]. At present, the research on MU is based on MRI measurement. Although prostate MRI is the most common imaging method, it has some limitations and deviations in measuring MU. Therefore, we used holographic imaging technology to reconstruct a new high-precision 3D dynamic model using unique technology from the data obtained from prostate MRI. This model can be rotated scaling, transparency, erasing of any organ, combination, splitting, and other operations. The surgeon can further clarify the location, size, the shape of the apex of the prostate, the shape of the base of the prostate, the position of the bladder neck, and other important information about prostate cancer, clarify the relationship between the prostate and surrounding blood vessels, and make proper preoperative planning. Its intuitive and 3D image can also be fused with the endoscopic image of laparoscopic surgery for the reference of surgeons to better avoid damage to the surrounding important organs and structures during the operation and complete the operation more accurately. In addition, we use the reconstructed 3D model to measure the MU parameters of patients, which can obtain more intuitive and accurate data. The research results are more accurate than the two-dimensional MRI data. Nowadays, relevant research has yet to be published. The 3D model we reconstructed can show the structure of the entire MU complex. We can also calculate the volume of the complex with unique technology, so we creatively proposed that MUV can also affect the recovery of postoperative UC of patients. We performed this study research to verify it.

Our research results show that patients with larger MUV and longer MUL have better UC immediately after catheter removal, consistent with our experimental expectations. Among the analysis results of achieving UC 1 month after RARP, patients with younger age, lower VPSS score, larger eMUV volume, and longer eMUL had better recovery of UC in 1 month after RARP. The results suggest that patients with eMUL > 13 mm have a higher probability of recovering social continence 1 month after RARP, consistent with the existing research results. The results of achieving UC in 3 months after RARP showed that the larger the MUV, the greater the probability of achieving UC in 3 months after RARP. In univariate analysis, the age factor has statistical significance in the patients achieving continence 1 month after RARP group and in the patients achieving continence 3 months after RARP group, consistent with the current research conclusion. In contrast, it has no statistical significance in the patients achieving continence immediately after removing catheter group, which we think the sample size of different groups causes the difference. We also performed a subanalysis that there were no statistically significant differences in MUL, MUV, and other parameters in different age range, making the research results of this article more convincing. The VPSS score was statistically significant only in the patients achieving continence 1 month after RARP group. The multivariate analysis suggested that it was an independent predictor of the recovery of UC 1 month after RARP. There was no statistical significance in other groups, which may be related to the difference caused by the subjective VPSS score. There is currently controversy over the impact of VPSS or IPSS on postoperative urinary continence after RARP, and the study did not yield our expected results may be due to uneven grouping and subjective scoring. A larger sample study is needed to demonstrate the relationship between LUTS and postoperative urinary continence after RARP. Through multivariate analysis, it is concluded that eMUV is a predictive factor for the early postoperative recovery of UC. Our results show that patients with better UC recovery had larger MUV and longer MUL than those with poor UC recovery, which showed a positive trend and was consistent with our expectations. The sample size may not be large enough, so no statistically significant results have been obtained from the current data.

Our study has limitations, including small sample size, single institution study, and lack of reconstruction of a 3D model to measure MU for postoperative MRI of patients. Whether the MUL of patients after surgery is the same as the eMUL reconstructed before surgery remains to be discussed. In future research, we will increase the sample size to analyze the data results. We will include the data of patients undergoing RARP from more institutions for multi-center research.

## Conclusion

Our study shows that those with larger MUV and longer MUL have better early postoperative UC recovery, further confirming MU's influence on postoperative UC recovery. Age and VPSS score may predict postoperative UC recovery, but more data are needed for further study. Compared with the MUL, the eMUV, independently associated with early postoperative UC recovery, is a better predictor.

## Data Availability

The data underlying this article will be shared on reasonable request to the corresponding author.
